# Reference ranges and determinants of total hCG levels during pregnancy: the Generation R Study

**DOI:** 10.1007/s10654-015-0039-0

**Published:** 2015-05-12

**Authors:** Tim I. M. Korevaar, Eric A. P. Steegers, Yolanda B. de Rijke, Sarah Schalekamp-Timmermans, W. Edward Visser, Albert Hofman, Vincent W. V. Jaddoe, Henning Tiemeier, Theo J. Visser, Marco Medici, Robin P. Peeters

**Affiliations:** The Generation R Study Group, Erasmus Medical Center, Rotterdam, The Netherlands; Department of Internal Medicine, Erasmus Medical Centre, Room D 430, Dr Molewaterplein 50, 3015 GE Rotterdam, The Netherlands; Rotterdam Thyroid Center, Erasmus Medical Center, Rotterdam, The Netherlands; Department of Clinical Chemistry, Erasmus Medical Center – Sophia Children’s Hospital, Rotterdam, The Netherlands; Department of Obstetrics and Gynecology, Erasmus Medical Center – Sophia Children’s Hospital, Rotterdam, The Netherlands; Department of Epidemiology, Erasmus Medical Center, Rotterdam, The Netherlands; Department of Epidemiology Pediatrics, Erasmus Medical Center – Sophia Children’s Hospital, Rotterdam, The Netherlands; Department of Child and Adolescent Psychiatry, Erasmus Medical Center – Sophia Children’s Hospital, Rotterdam, The Netherlands

**Keywords:** hCG, Pregnancy, Reference range, Determinants

## Abstract

Human chorionic gonadotropin (hCG) is a pregnancy hormone secreted by the placental synctiotrophoblast cell layer that has been linked to fetal growth and various placental, uterine and fetal functions. In order to investigate the effects of hCG on clinical endpoints, knowledge on reference range (RR) methodology and determinants of gestational hCG levels is crucial. Moreover, a better understanding of gestational hCG physiology can improve current screening programs and future clinical management. Serum total hCG levels were determined in 8195 women participating in the Generation R Study. Gestational age specific RRs using ‘ultrasound derived gestational age’ (US RRs) were calculated and compared with ‘last menstrual period derived gestational age’ (LMP RRs) and a model-based RR. We also investigated which pregnancy characteristics were associated with hCG levels. Compared to the US RRs, the LMP RRs were lower, most notably for the median and lower limit levels. No considerable differences were found between RRs calculated in the general population or in uncomplicated pregnancies only. Maternal smoking, BMI, parity, ethnicity, fetal gender, placental weight and hyperemesis gravidarum symptoms were associated with total hCG. We provide gestational RRs for total hCG and show that total hCG values and RR cut-offs during pregnancy vary depending on pregnancy dating methodology. This is likely due to the influence of hCG on embryonic growth, suggesting that ultrasound based pregnancy dating might be less reliable in women with high/low hCG levels. Furthermore, we identify different pregnancy characteristics that influence total hCG levels considerably and should therefore be accounted for in clinical studies.

**Electronic supplementary material:**

The online version of this article (doi:10.1007/s10654-015-0039-0) contains supplementary material, which is available to authorized users.

## Introduction

Human chorionic gonadotropin (hCG) is a pregnancy hormone secreted by the placental synctiotrophoblast cell layer. hCG levels have a very typical trajectory during pregnancy. hCG levels increase exponentially during very early pregnancy, after reaching a plateau during the late first trimester hCG levels steadily decline until a steady state which is seen throughout the second and third trimesters. Classically, hCG is known for maintaining the corpus luteum and its progesterone production, which is essential for embryo implantation [1–3]. Various types of studies have linked hCG to other placental, uterine and fetal functions such as umbilical cord development, suppression of myometrial contractions, the promotion of growth and differentiation of fetal organs but also angiogenesis and regulation of immune tolerance [4]. Although the main clinical utility of hCG levels lies within early pregnancy, these findings underline the importance of hCG throughout gestational physiology and suggest that variations in hCG levels may be associated with adverse clinical outcomes.

Indeed, abnormal levels of hCG have previously been associated with adverse pregnancy outcomes such as fetal loss, preeclampsia, preterm delivery and fetal growth restriction [5–10]. In order to study such clinical associations, it is essential to establish correct gestational age-dependent reference ranges (RRs) which can be difficult because hCG itself has been proposed as a marker of gestational age [11]. hCG has been shown to be and to determine confounding and mediating factors such as differences between different measurement methodologies, pregnancy dating methodologies and differences in population characteristics [12–15]. The latter is especially important because previous studies have demonstrated that certain maternal or fetal characteristics, such as maternal smoking, parity, ethnicity, body-mass index (BMI), placental weight, hyperemesis gravidarum symptoms and fetal gender, that are associated with an increased risk of adverse pregnancy outcomes, are also associated with hCG levels [16–23].

This study aims to identify determinants of hCG levels during pregnancy that play a role in the complex relationship between hCG and clinical outcomes. We investigated in a large prospective-based cohort study the difference between RRs calculated according to pregnancy dating by ultrasound (US RRs) and RRs determined according to last menstrual period (LMP RRs). In addition, we compared reference range determination by a sensitive model-based approach with the more conventional non-parametric approach and studied if total hCG RRs determined in the general population are different from RRs calculated in uncomplicated pregnancies only. Furthermore, we analyzed which maternal and fetal characteristic are associated with total hCG levels.

## Materials and methods

### Study population

This study was embedded in the Generation R Study, a population-based prospective cohort from early fetal life onwards in Rotterdam, The Netherlands [24].

In 8195 pregnant women, total serum hCG levels were determined from blood samples drawn from the women at inclusion in the study (median 14.4 weeks; 95 % range 10.1–26.2). Women with a late termination of pregnancy (TOP) were excluded from the study population (n = 2). For population-based RR, and total hCG determinant analyses, women with twin pregnancies (n = 90) or in vitro fertilization treatment (n = 38) were excluded (Supplemental Table 5).

### Serum measurements

hCG was analyzed in serum using a solid-phase two-site chemiluminescent immunometric assay, calibrated against WHO 3rd IS 75/537, on an Immulite 2000 XPi system (Siemens Healthcare Diagnostics, Deerfield, IL, USA). The Siemens assay detects serum intact hCG, hyperglycosylated hCG, serum nicked hCG, serum nicked hyperglycosylated hCG, serum asialo hCG, serum hCG free β-subunit and serum nicked hCG β [25]. The inter assay coefficient of variation was 8.0, 6.3 and 5.1 % at the concentration of 9.7, 53.1 and 821.5 IU/L, respectively. Although the Immulite 2000 is considered as one of the best assays for total hCG, it should be noted that the reference ranges in this paper are assay specific and do not correspond with hCG values obtained from different assays [26].

### Covariates

Ultrasound examinations were performed using an Aloka^®^ model SSD-1700 (Tokyo, Japan) or the ATL-Philips^®^ Model HDI 5000 (Seattle, WA, USA). Fetal biometry consisting of BPD (outer–outer), HC, TCD, AC and FL was measured during each ultrasound examination. CRL was measured in early pregnancy if feasible and Verburg’s equation was used to transform CRL to gestational age [27]. CRL was measured in a true mid-sagittal plane with the genital tubercle and the fetal spine longitudinally in view. The maximum length from cranium to the caudal rump was measured as a straight line. BPD and HC were measured in a transverse section of the head with a central midline echo, interrupted in the anterior third by the cavity of the septum pellucidum with the anterior and posterior horns of the lateral ventricles in view. For BPD the outer–outer diameter was measured perpendicular to the midline and for HC an ellipse was drawn around the outline of the skull. For the TCD measurement the transducer was rotated from the transverse plane for measurement of the BPD towards the cerebellum in the back of the head while keeping the cavity of the septum pellucidum in view. The optimal plane was reached when the peduncles were visualized with a symmetrical shaped cerebellum. The calipers were placed on the outer, lateral edges of the cerebellum. AC was measured in a symmetrical, transverse, round section through the abdomen, with visualization of the vertebrae on a lateral position in alignment with the ribs. The measurement was taken in a plane with the stomach and the bifurcation of the umbilical and hepatic veins using an ellipse around the abdomen. FL was measured with the full length of the bone in view perpendicular to the ultrasound beam. Transvaginal scanning was performed in case of limited visibility by transabdominal scanning in early pregnancy.

Quality checks were carried out frequently to assess the correctness of the ultrasound sections used for biometry measurements and placements of the calipers. Feedback was provided when needed to optimize individual performance. As experience in early pregnancy is limited, intraobserver and interobserver reproducibility of fetal ultrasound measurements from 9 to 14 weeks of gestation was assessed in 21 pregnancies. The intraclass correlation coefficient (ICC) and coefficient of variation (CV) were calculated. The ICC was higher than 0.98 and the corresponding CV lower than 6 % for all fetal biometry parameters. Bland and Altman plots to test agreement of measurements for fetal biometry demonstrated normal distributions; the mean difference was around zero and 95 % of measurements fell within 2SD of the mean. The 95 % limits of agreement for differences in fetal biometry measurements between and among operators in proportions fell within 10 % of the mean of the measurements, indicating good reproducibility [27].

Last menstrual period (LMP) was obtained from the referring letter from the community midwife or hospital. This date was confirmed with the mother at the ultrasound visit and additional information on the regularity and cycle duration was obtained. A subset of 2948 women included during early pregnancy were selected for ascertainment of LMP gestational age, subsequently women with neither a known first day of the last menstrual period nor a regular menstrual cycle of 28 plus or minus 4 days were excluded (n = 1431). In case of a discrepant result between the LMP obtained from hospital/midwife letters and self-reported LMP at the research center, the LMP closest to the gestational age based on CRL measurement was used. Information on maternal age, parity, ethnicity, education and smoking status was obtained by questionnaires during pregnancy. Information on fertility treatment, mode of delivery, pregnancy outcome, date of birth, birth anthropometrics, and child gender were obtained from community midwives, obstetricians, and hospital registries [24].

### Statistical analysis

Non-parametric gestational age specific RRs were determined by the 2.5th–97.5th percentiles for each gestational week. In order to compare total hCG values throughout gestation, multiple of median (MoM) values were calculated by dividing each participant’s total hCG level with the median value of the total group for that particular gestational week. Model-based reference ranges were created using Generalized Additive Models for Location, Size and Shape (GAMLSS). These specific statistical tools enable flexible, (semi) parametric, RR calculations while accounting for skewness and kurtosis of the data during the modelling process. We used 15 cubic splines for gestational age at blood sampling, 3 cubic splines for sigma variation and a Box Cox *t* family distribution (after sensitivity analyses using Akaike Information Criterion and worm plots) in order to achieve the best fit, while also accounting for the known, typical pregnancy hCG trajectory [28]. Subsequently, gestational age specific Z-scores were derived from the model. In order to compare the model cut-off values to the non-parametric cut-off values (calculated per week), 2.5th, 50th and 97.5th values calculated for the middle of each week were derived from the model.

Because hCG may influence early fetal growth, gestational age that is defined according to fetal growth (US RRs) may differ according to hCG levels. For this reason, we also defined gestational age according to the first day of the LMP in a subgroup of mothers with data available on LMP that had a regular menstrual cycle (28 plus or minus 4 days; n = 1526) [29, 30].

As hCG levels may differ in complicated pregnancies, RRs were also determined in uncomplicated pregnancies only. For these analyses we selected women with uncomplicated pregnancies by excluding pregnancies with a non-live born child, preterm birth, a small for gestational age newborn, hypertensive disorders or pre-existing hypertension, resulting in a population of n = 7015; definitions of complicated pregnancies have previously been described in detail [31–33].

Since hCG is secreted by trophoblasts, the number of trophoblast cells (approximated by the weight of the placenta) may influence total hCG levels. Therefore, we investigated whether placental weight at birth is associated with total hCG MoM levels. Furthermore, it is speculated that hCG plays a role in hyperemesis gravidarum, and therefore we investigated if specific hyperemesis gravidarum symptoms (reflux/belching, nausea or vomiting) are associated with total hCG MoM levels.

For covariates with missing data, multiple imputation according to the Markov Chain Monte Carlo method was used [34]. Five imputed data sets were created and pooled for analyses. Maternal smoking, education, ethnicity, BMI, parity and child gender were added to the model (missing due to non-response in 12.6, 9.0, 5.4 and <2 %, respectively). Furthermore, we added gestational age at time of blood sampling, maternal age, and pregnancy complications as prediction variables only. No significant differences in descriptive characteristics were found between the original and imputed datasets. Confidence intervals for US RRs were created using bootstrap analyses with 1000 sample draws. The associations between maternal or fetal characteristics and total hCG (MoM) levels were analyzed by ANOVA and linear regression. Univariate analyses were adjusted for gestational age at blood sampling and multivariate analyses were adjusted for gestational age at blood sampling, maternal age, smoking, BMI, education level, maternal ethnicity, parity and child gender. To achieve normal distribution for statistical testing, total hCG values and MoM values were transformed by the natural logarithm. The above analyses were performed using Statistical Package of Social Sciences version 21.0 for Windows (SPSS Inc. Chicago, IL, USA). The associations between pregnancy characteristics and total hCG MoM levels depicted in the figures were assessed by ordinary least squares fitting functions with restricted cubic splines from the RMS library in R statistical package, version 3.03.

## Results

**Fig. 1:**
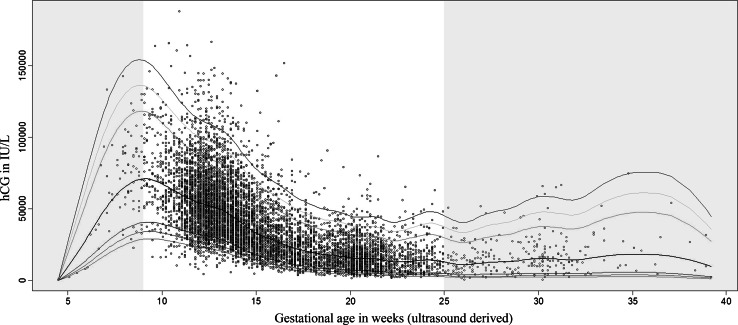
Gestational age specific reference ranges for total hCG levels during pregnancy. Total hCG reference range values were calculated according to a (semi) parametric 2.5th–97.5th percentiles by GAMLSS modelling in a population-based approach among the entire study population, after exclusion of women with IVF treatment (N = 38), twin pregnancy (N = 90) or TOP pregnancies (N = 2). Colored lines depict the gestational age specific centiles for total hCG levels. Grey area depict areas with higher uncertainty due to small numbers (N per week <40 before week 9 and after week 24)

Descriptive characteristics of the study population are shown in Supplemental Table 1. Population-based, gestational age specific median and RR values for total hCG are shown in Table 1 and model-based reference centile curves are depicted in Fig. 1. Throughout gestation, total hCG levels showed a peak in the 9th and 10th week of gestation, after which a steady decline was observed.

**Table 1:** Gestational age specific, total population reference ranges for hCG in 8065 women

Gestational week	N	Median	Minimum	2.5th	97.5th	Maximum
<9	32	59.973	455	2.305	94.251	142.584
9	50	75.494	22.655	24.310	125.882	129.909
10	106	74.655	16.080	24.370	137.697	163.393
11	255	62.493	10.340	23.669	129.242	187.852
12	790	56.004	8.105	22.846	114.774	164.125
13	1.418	52.367	4.618	23.272	109.990	166.478
14	1.069	47.267	5.925	20.494	105.369	144.054
15	800	37.303	4.834	14.262	82.506	122.037
16	594	29.614	7.512	11.159	80.656	132.084
17	455	24.426	5.637	8.294	69.447	151.558
18	354	20.693	3.822	6.637	50.109	75.993
19	271	17.609	3.895	5.022	52.640	90.628
20	389	17.354	3.128	5.342	43.692	78.841
21	530	15.088	1.542	4.213	42.892	73.485
22	330	16.174	2.559	3.689	44.548	86.541
23	165	12.415	1.957	2.390	43.379	65.192
24	134	13.739	2.511	4.067	45.031	49.392
25	79	14.749	3.354	3.847	53.383	63.166
>25	244	13.852	518	2.228	58.125	74.719

hCG reference range values were calculated according to a population-based approach in the whole study population, after exclusion of women with IVF treatment (N = 38), twin pregnancy (N = 90) or TOP pregnancies (N = 2)

### Reference range comparisons

Pregnancy dating based on ultrasound is determined by fetal size. Considering that hCG is associated with fetal growth, we studied if gestational age specific hCG RRs are different when gestational age is determined by ultrasound (US RRs) or based on the first day of the last menstrual period (LMP RRs). As is shown in Table 2, compared to US RRs, LMP RR levels showed a shift to the left with particularly lower levels for the median and lower limit levels. For RRs determined in women with an uncomplicated pregnancy, only small differences with the population-based approach were seen (Supplemental Table 2).

**Table 2:** Comparison of reference ranges for total hCG according to gestational age determined by ultrasound or last menstrual period (LMP)

Gestational week	N Ultrasound	*N LMP*	Median hCG (95% CI)		*LMP*	2.5th percentile (95% CI)		*LMP*	97.5th percentile (95% CI)		*LMP*
11	255	*91*	62.493	(58.665–67.327)	*56.780*	23.669	(16.372–26.937)	*11.189*	129.242	(111.434–160.438)	*132.875*
12	790	*470*	56.004	(54.242–58.142)	*52.252*	22.846	(19.793–24.392)	*12.193*	114.774	(110.101–126.943)	*110.118*
13	1.418	*633*	52.367	(51.237–53.893)	*50.596*	23.272	(21.953–25.260)	*14.547*	109.990	(103.844–116.031)	*105.402*
14	1.069	*323*	47.267	(45.697–48.706)	*47.965*	20.494	(17.626–21.988)	*12.842*	105.369	(96.283–110.567)	*96.874*

*Italic numbers* = gestational age determined by reliable first day of last menstruation. hCG reference range values were calculated according to a population-based approach in the whole study population, after exclusion of women with IVF treatment (N = 38), twin pregnancy (N = 90) or TOP pregnancies (N = 2). Gestational age at blood sampling was determined according to ultrasonography measured crown-rump length or first day of last menstrual period, if reliable. 95CIs were determined by bootstrap analyses using 1000 sample draws

Supplemental Table 3 shows the median, and upper or lower limit cut-off values for total hCG as calculated by the previous non-parametric method compared to the same cut-off values derived from a model-based approach. In general, the model-based RRs were in the low-normal region of the non-parametric RRs 95 % confidence interval. However, overall there was not a statistically significant differences between the cut-off values from both methods. Furthermore, the z-scores derived from the model were highly correlated with the commonly used Multiple of Median (MoM) values (Standardized β = 0.919; data not shown).

### Determinants of hCG

**Fig. 2:**
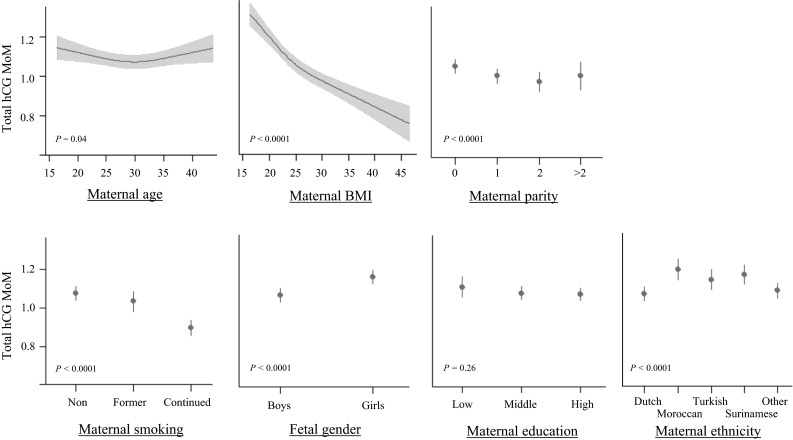
The relationship between maternal or fetal characteristics and total hCG MoM levels. Plots show the relationship between pregnancy characteristics and total hCG MoM levels for continuous and categorical variables as predicted mean with 95 % confidence interval. Analyses were performed after exclusion of women with IVF treatment (N = 38), twin pregnancy (N = 90) or TOP pregnancies (N = 2), and were adjusted for maternal age, smoking, BMI, parity, education level, ethnicity and fetal gender

Figure 2 shows the association between maternal or fetal characteristics and total hCG levels adjusted for gestational week by multiple of median (MoM) transformation. Taken together, the determinants depicted explained 6.7 % of the variability with maternal smoking, BMI, parity and child gender as the main determinants of total hCG (MoM) levels. Compared to non-smokers, smokers on average had lower total hCG values (−6.299 ± 642 IU/L; *P* < 0.001) and the effects of smoking on total hCG levels were dose dependent. The effect of smoking on total hCG levels was modified by gestational age (interaction term ‘smoking(yes)’ * ‘gestational age at blood sampling’: *P* = 0.10; with corresponding β for total hCG MoM level for the first, second (wk 13.1–16.5) and third tertile of gestational age of −0.143, −0.189 and −0.186, respectively). The total hCG values of women who stopped smoking after a positive pregnancy test were similar to non-smokers. Women within the highest BMI quintile on average had a substantially lower mean total hCG level compared to women within the first quintile (average difference 9369 ± 729 IU/L, *P* < 0.001; Supplemental Table 4) and mean total hCG level differences according to parity and child gender ranged between approximately 2000–4000 IU/L. These results remained similar after multivariate correction for potential confounders (Supplemental Table 4). We also investigated the women who were excluded for these analyses and found that IVF treatment and twin pregnancies were associated with higher mean total hCG (MoM) levels (Supplemental Table 5).

**Fig. 3:**
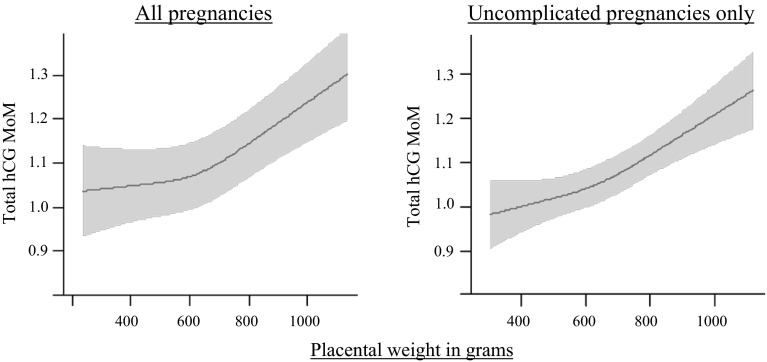
The relationship between placental weight and total hCG MoM levels. Plots show the relationship between placental weight at birth and total hCG MoM levels as predicted mean with 95 percent confidence interval. Analyses were performed after exclusion of women with IVF treatment (N = 38), twin pregnancy (N = 90) or TOP pregnancies (N = 2; placental weight available in n = 5851) and were adjusted for maternal age, smoking, BMI, parity, education level, ethnicity and fetal gender. For uncomplicated pregnancies we selected women’s first pregnancy registered in our database and excluded pregnancies with a non-live born child, preterm birth, a small for gestational age newborn, hypertensive disorder or pre-existing hypertension resulting in a population of n = 7015 (with placental weight available n = 4999)

As is shown in Fig. 3, an increase in placental weight was associated with an increase in total hCG MoM values. In the multivariate model, placental weight remained associated with total hCG levels. Although addition of placental weight to the model did reduce the strength of the associations between BMI, smoking, parity, ethnicity or fetal gender and total hCG (MoM) levels, these associations remained highly significant. Furthermore, an increasing frequency of self-reported hyperemesis gravidarum symptoms (i.e. reflux/belching, nausea or vomiting) was associated with an increase in total hCG MoM values (Supplemental Table 6).

## Discussion

Total hCG values and RR cut-offs during pregnancy vary depending on different methodological as well as individual factors. In the current study we determined a population-based gestational age specific RR for total hCG during pregnancy and we demonstrate that these RRs differ depending on the methodology used to determine gestational age. Furthermore, we show that maternal smoking, BMI, parity, ethnicity, child gender and placental weight are factors associated with total hCG levels and that increasing severity of reflux/belching, nausea and vomiting symptoms was associated with increasing total hCG levels.

We determined RRs for total hCG amongst the whole population and when we compared such RRs with RRs calculated in women with uncomplicated pregnancies we found only small, negligible differences. RRs were also calculated using a model-based approach. Although there was an overall trend for lower estimates as compared to the non-parametric methods, these differences overall did not reach statistical significance. Future analyses should determine whether these differences in cut-off values influence the associations of total hCG with pregnancy complications or whether there are consequences for the identification of women with a clinically relevant increased risk of other adverse outcomes. However, considerable differences were present between the US RRs and the LMP RRs. Overall, US RRs were higher compared to LMP RRs and as such it seems likely that US RRs are affected by the effects of hCG on fetal growth. This fits with observations that hCG levels are negatively associated with fetal growth [35, 36]. Moreover, this suggests that pregnancy dating by ultrasound, which is considered the gold standard, might be less reliable in women with relatively high or low levels of hCG.

We show that BMI is one of the most influential determinants of total hCG levels, exhibiting an inverse association. Previous studies have shown a similar association between hCG and BMI, and some aneuploidy screening programs use BMI corrected values in order to increase testing performance [18, 19, 37]. The pathophysiology behind these associations is currently unclear. BMI has been positively associated with placental weight and increasing placental weight is associated with increasing hCG levels in this study. This may suggest that higher placental weight in women with high BMI levels may compensate the negative association between BMI and hCG. However, in a subset of women in which placental weight was known (n = 5851), the association between BMI and total hCG MoM levels remained similar after adjustment for placental weight (β ± SE per ln(MoM) change; unadjusted: −0.019 ± 0.001 vs. adjusted: −0.020 ± 0.001; data not shown) suggesting separate mechanisms in the effects on hCG. The pathways via which this effect occurs remain to be elucidated and a potential role for adipokines or inflammatory markers should be considered [38–40].

Similar to previous studies, smoking was associated with lower hCG levels in the current study as well. However, we are the first to show that women who stopped smoking when the pregnancy test was positive had similar total hCG levels as non-smokers (Supplemental Table 4). This indicates that discontinuation of smoking at the time of known pregnancy may prevent the reduction in total hCG levels seen amongst continuing smokers and that the effects of smoking on total hCG levels will only become apparent after a particular smoking duration (dose dependency). Indeed, similar to findings by Ball et al. [41, 42], the strength of the association between total hCG and smoking increased with gestational age. Most likely, this effect is a cumulative smoking effect considering that we also found a strong dose-dependent association between the number of cigarettes smoked and total hCG decrease. For aneuploidy screening, usually utilizing β-hCG levels, neither the total effects of smoking nor the gestational age dependent effects had a considerable impact on the outcome [16, 41, 43]. Prenatal smoking has consistently been associated with an increased risk of small for gestational age children and low placental weight. It is likely that the effects of prenatal smoking on birth weight of the newborn are at least in part caused by a decrease in hCG levels as it has been shown that prenatal smoking leads to an increase in apoptosis of synctiotrophoblast cell layer [44]. Future studies should investigate to what extent hCG contributes to the changes in fetal growth and birth weight. Moreover, given the unequivocal link between smoking and adverse perinatal outcomes, the strong association between smoking and total hCG levels is a clear demonstration of the confounding potential of pregnancy characteristics in studies investigating the relationship between hCG levels and any clinical outcomes/measurements.

Interestingly, in particular the effects of smoking, but also the effects of other characteristics seemed to be more pronounced in our study compared to other studies [16–18, 20, 21, 43, 45]. This may be due to the fact that we determined total hCG levels using an assay which detects the vast majority of hCG variants [25] whereas most other studies report the effects on β-hCG. In turn, this could suggest that BMI, smoking, parity, ethnicity, child gender and placental weight have differential effects on specific types of hCG such as nicked or hyperglycosylated hCG.

To our knowledge, this is the only study which reports RRs for total hCG during pregnancy apart from the manufacturer of the assay that we used, which reported on 593 pregnant women [46]. Furthermore, we are the first to report the associations between detailed maternal and fetal characteristics and total hCG levels during pregnancy. Access to an extensive database allowed us to compare different methods of RR determinations and study the association of various sparsely reported maternal/pregnancy characteristics including placental weight and vomiting symptomatology. We were, however, limited by the fact that LMP and the menstrual cycle, placental weight and vomiting symptoms were only available in a subset of women. Also, the number of women with availability of total hCG measurements varied for each gestational week and therefore reference range determinations were not equally reliable throughout gestation, particularly during very early and the third trimester of pregnancy. Potential differences in formulas used to determine gestational age based on ultrasound data may also underlie some of our results and warrant further research.

In conclusion, we provide data on total hCG reference ranges during pregnancy from a large prospective population-based cohort and identified that these may considerably differ according to pregnancy dating methodology. Furthermore, we found that total hCG differs according to maternal BMI, smoking, parity, ethnicity, child gender, placental weight and hyperemesis gravidarum symptoms. Our results suggest that the association between gestational age, hCG and fetal growth can cause less reliable ultrasound derived pregnancy dating, in particular in women with high or low levels of hCG. These data underline the complex relations between hCG, maternal and fetal factors, which should be taken into account when studying pregnancy complications. Our findings can serve as a reference for various clinical research studies and warrant further research on reference range determination for hCG during pregnancy.

## Electronic supplementary material

Supplementary material 1 (DOCX 44 kb)
