# Supplementary material for: Reference ranges and determinants of total hCG levels during pregnancy: the Generation R Study
Source: Eur J Epidemiol. 2015 May 12;30(9):1057–66. doi: 10.1007/s10654-015-0039-0 (PMC4584104; doi:10.1007/s10654-015-0039-0)
Supplement: Supplementary file 1 — Supplementary material 1 (DOCX 44 kb) [file 10654_2015_39_MOESM1_ESM.docx]

| **Supplemental Table 1** Descriptive characteristics of the study population. | | | | | | | | |
| --- | --- | --- | --- | --- | --- | --- | --- | --- |
|  | |  | **Median or  N per group** | | **(95% range)** | | |  |
|  | | |  |  | | | |  |
| hCG (IU/L) | | | 35,403 | (6,074– 100,351) | | | |  |
| **MoM** | | | 1.00 | (0.33 – 2.46) | | | |  |
| hCG z-scores (SD) | | | 0.00 | (-1.95 - +1.97) | | | |  |
|  | | |  |  | | | |  |
| **Gestational age** (weeks) | | |  |  | | | |  |
| Ultrasound | | | 14.4 | (10.1 – 26.1) | | | |  |
| Last menstrual period^a^ | | | 12.4 | (10.7 – 12.4) | | | |  |
|  | | |  |  | | | |  |
| **Maternal age** (years) | | | 30.2 | (19.3 - 39.1) | | | |  |
|  | | |  |  | | | |  |
| **BMI** (kg/m^2^) | | | 23.9 | (18.7 – 36.5) | | | |  |
|  | | |  |  | | | |  |
| **Placental weight** (g) | | | 620 | (390 – 950) | | | |  |
|  | | |  |  | | | |  |
| Parity | | |  |  | |  | |  |
| 0 | | | 55.4 | (4469) | |  | |  |
| 1 | | | 30.0 | (2417) | |  | |  |
| 2 | | | 10.4 | (834) | |  | |  |
| >2 | | | 4.2 | (339) | |  | |  |
| Smoking | | |  |  | |  | |  |
| Non-smokers | | | 2874 | (74.9) | |  | |  |
| Stopped smokers | | | 381 | (9.9) | |  | |  |
| Smokers | | | 584 | (15.2) | |  | |  |
| Education level | | |  |  | |  | |  |
| Low | | | 12.5 | (1013) | |  | |  |
| Middle | | | 47.2 | (3805) | |  | |  |
| High | | | 40.3 | (3247) | |  | |  |
| **Ethnicity** | | |  |  | |  | |  |
| Dutch | | | 48.1 | (3881) | |  | |  |
| Moroccan | | | 7.3 | (591) | |  | |  |
| Turkish | | | 9.7 | (779) | |  | |  |
| Surinamese | | | 9.2 | (745) | |  | |  |
| Other | | | 25.7 | (2069) | |  | |  |
|  | | |  |  | |  | |  |
| **Child gender** (boys %) | | | 4062 | (50.4) | |  | |  |
| ^a^Available in a subset of 1517 women included during early pregnancy.  ^b^ Available in a subset of 5822 women. | | | | | |  |  |  |

| **Supplemental Table 2.** Gestational age specific, reference ranges for hCG in 7015 women with an uncomplicated pregnancy. | | | | | | | |
| --- | --- | --- | --- | --- | --- | --- | --- |
| **Gestational week** | **N** | | **Median** | **Minimum** | **2.5th** | **97.5th** | **Maximum** |
| **<9** | 27 | 59.973 | | 455 | 2.305 | 93.158 | 142.584 |
| **9** | 40 | 78.705 | | 22.655 | 22.906 | 129.808 | 129.909 |
| **10** | 91 | 73.369 | | 16.464 | 29.794 | 133.550 | 145.910 |
| **11** | 221 | 61.794 | | 16.151 | 23.806 | 133.221 | 187.852 |
| **12** | 678 | 56.692 | | 8.105 | 23.807 | 114.394 | 144.919 |
| **13** | 1255 | 53.064 | | 4.618 | 23.119 | 108.752 | 166.478 |
| **14** | 945 | 47.406 | | 5.925 | 21.172 | 102.380 | 144.054 |
| **15** | 695 | 37.240 | | 4.834 | 14.641 | 83.065 | 122.037 |
| **16** | 517 | 29.344 | | 7.512 | 11.163 | 80.056 | 132.084 |
| **17** | 387 | 23.988 | | 5.999 | 8.235 | 67.195 | 142.918 |
| **18** | 308 | 20.843 | | 3.822 | 6.763 | 50.777 | 75.993 |
| **19** | 233 | 17.609 | | 3.895 | 4.544 | 49.569 | 90.628 |
| **20** | 339 | 16.855 | | 3.128 | 5.020 | 44.217 | 57.091 |
| **21** | 467 | 14.825 | | 1.542 | 4.237 | 42.196 | 73.485 |
| **22** | 281 | 16.355 | | 2.810 | 3.740 | 44.717 | 86.541 |
| **23** | 145 | 12.715 | | 1.957 | 2.336 | 38.060 | 48.059 |
| **24** | 115 | 13.177 | | 2.511 | 3.974 | 46.470 | 49.392 |
| **25** | 68 | 16.465 | | 3.354 | 3.711 | 56.073 | 63.166 |
| **>25** | 203 | 13.503 | | 518 | 2.224 | 53.749 | 74.719 |
| hCG reference range values were calculated in women with an uncomplicated pregnancy, after exclusion of women with IVF treatment (N=38), twin pregnancy (N=90) or TOP pregnancies (N=2). Women that gave birth to a live-born, singleton pregnancy which was not premature (<37^th^ week), small for gestational age (<2.5^th^ gestational age specific birth weight), and of which the pregnancy was not complicated by pre-existing or pregnancy-induced hypertension or preeclampsia were considered uncomplicated. In addition, only the first recorded pregnancy within our study was used. | | | | | | | |

| **Supplemental Table 3.** Comparison of reference ranges of model-based versus non-parametric approach in 8065 women. | | | | | | | | | | | | | |  |
| --- | --- | --- | --- | --- | --- | --- | --- | --- | --- | --- | --- | --- | --- | --- |
|  | **Gestational week** | **N** | **Model median** | **Non-parametric median 95% CI** | |  | **Model 2.5^th^** | **Non-parametric 2.5^th^ 95% CI** | |  | **Model 97.5^th^ p** | **Non-parametric 97.5^th^ 95% CI** | | |
|  |  |  |  | *Lower* | *Upper* |  |  | *Lower* | *Upper* |  |  | *Lower* | *Upper* | |
|  | **9** | 50 | 69.204 | 64.445 | 86.851 |  | 27.702 | 22.655 | 34.928 |  | **151.958** | **115.695** | **129.909** | |
|  | **10** | 106 | 70.180 | 69.350 | 81.789 |  | 29.323 | 16.080 | 35.517 |  | 149.276 | 124.030 | 163.393 | |
|  | **11** | 255 | 63.848 | 58.665 | 67.776 |  | 27.358 | 16.372 | 27.634 |  | 133.260 | 111.836 | 160.438 | |
|  | **12** | 790 | 56.681 | 54.420 | 58.079 |  | **24.478** | **19.136** | **24.382** |  | 117.604 | 110.101 | 126.782 | |
|  | **13** | 1.418 | 52.152 | 50.969 | 53.938 |  | 22.231 | 21.744 | 25.231 |  | 109.276 | 103.691 | 116.644 | |
|  | **14** | 1.069 | 47.015 | 45.492 | 48.706 |  | 19.231 | 17.801 | 21.972 |  | 101.601 | 96.478 | 110.567 | |
|  | **15** | 800 | 37.478 | 36.055 | 39.115 |  | 14.314 | 12.719 | 15.322 |  | 85.173 | 78.800 | 89.593 | |
|  | **16** | 594 | 30.220 | 28.491 | 30.887 |  | 10.630 | 9.881 | 12.134 |  | 72.860 | 72.645 | 94.288 | |
|  | **17** | 455 | 25.106 | 23.409 | 26.182 |  | 8.178 | 7.232 | 9.485 |  | 63.878 | 63.839 | 75.868 | |
|  | **18** | 354 | 20.937 | 19.022 | 23.441 |  | 6.432 | 5.116 | 7.526 |  | 55.455 | 47.556 | 57.687 | |
|  | **19** | 271 | 18.379 | 16.315 | 19.764 |  | 5.417 | 3.998 | 6.321 |  | 50.064 | 41.000 | 64.266 | |
|  | **20** | 389 | 17.014 | 16.500 | 18.487 |  | 4.837 | 4.027 | 6.368 |  | **47.480** | **39.201** | **47.446** | |
|  | **21** | 530 | 15.429 | 13.838 | 16.282 |  | 4.189 | 3.379 | 4.689 |  | 44.380 | 38.682 | 47.894 | |
|  | **22** | 330 | **14.714** | **14.955** | **17.383** |  | 3.768 | 3.411 | 5.119 |  | 43.961 | 38.271 | 56.344 | |
|  | **23** | 165 | 13.084 | 10.829 | 15.145 |  | 3.133 | 2.153 | 3.332 |  | 40.805 | 34.138 | 55.899 | |
|  | **24** | 134 | 13.879 | 11.862 | 15.978 |  | 3.100 | 2.934 | 4.297 |  | 45.210 | 35.997 | 48.845 | |
|  | **25** | 79 | 14.047 | 11.753 | 18.961 |  | **2.933** | **3.354** | **4.598** |  | 47.684 | 36.803 | 63.166 | |
| Non-parametric total hCG reference range values were calculated according to the 2.5^th^ – 97.5^th^ percentiles, model-based cut-off values were calculated by (semi) parametric GAMLSS modelling, both references were derived by population-based approach in the whole study population, after exclusion of women with IVF treatment (N=38), twin pregnancy (N=90) or TOP pregnancies (N=2). 95CIs were determined by bootstrap analyses using 1000 sample draws. | | | | | | | | | | | | | |  |

| **Supplemental Table 4.** Mean hCG values according to characteristics of 8065 women. | | | | | | |
| --- | --- | --- | --- | --- | --- | --- |
|  |  | | **Mean hCG** (IU/L) | |  |  |
|  | **% or range (N)** | | **Univariate** | **Multivariate** | **MoM hCG*** |  |
| Maternal age (years) |  | |  |  |  |  |
| 1^st^ quintile | 15-25 | (1630) | 41.261 | 40.971 | 1.13 |  |
| 2^nd^ quintile | 25-29 | (1623) | 39.856 | 39.869 | 1.08 |  |
| 3^rd^ quintile (ref) | 29-32 | (1616) | 40.698 | 40.482 | 1.10 |  |
| 4^th^ quintile | 32-34 | (1604) | 39.539 | 39.604 | 1.09 |  |
| 5^th^ quintile | 34-46 | (1591) | 38.982^a^ | 39.461 | 1.10 |  |
|  |  |  |  |  |  |  |
| Parity |  | |  |  |  |  |
| 0 (ref) | 55.4% | (4469) | 41.310 | 40.852 | 1.13 |  |
| 1 | 30.0% | (2417) | 39.004^c^ | 39.190^c^ | 1.08^c^ |  |
| 2 | 10.4% | (834) | 37.431^c^ | 38.591^c^ | 1.04^c^ |  |
| >2 | 4.2% | (339) | 38.130^b^ | 39.958 | 1.07 |  |
|  |  |  |  |  |  |  |
| **Ethnicity** |  | |  |  |  |  |
| Dutch (ref) | 48.1% | (3881) | 39.820 | 39.569 | 1.07 |  |
| Moroccan | 7.3% | (591) | 41.817^a^ | 42.209^c^ | 1.18^c^ |  |
| Turkish | 9.7% | (779) | 39.167 | 41.071^a^ | 1.14^c^ |  |
| Surinamese | 9.2% | (745) | 42.155 | 42.603^b^ | 1.16^c^ |  |
| Other | 25.7% | (2069) | 39.674 | 39.155 | 1.08 |  |
|  |  |  |  |  |  |  |
| **BMI** |  |  |  |  |  |  |
| 1^st^ quintile | 15-21 | (1626) | 45.709^c^ | 45.545^c^ | 1.23^c^ |  |
| 2^nd^ quintile | 21-23 | (1608) | 41.260^a^ | 41.240 | 1.14 |  |
| 3^rd^ quintile (ref) | 23-25 | (1604) | 39.231 | 39.321 | 1.10 |  |
| 4^th^ quintile | 25-28 | (1616) | 38.109^a^ | 38.082^a^ | 1.05^a^ |  |
| 5^th^ quintile | 28-51 | (1612) | 36.053^c^ | 36.176^c^ | 0.96^c^ |  |
|  |  |  |  |  |  |  |
| **Smoking** |  |  |  |  |  |  |
| No (ref) | 72.9% | (5880) | 41.309 | 41.278 | 1.13 |  |
| Former | 8.3% | (673) | 41.438 | 41.086 | 1.10 |  |
| Yes | 18.8% | (1512) | 34.703^c^ | 34.979^c^ | 0.96^c^ |  |
|  |  |  |  |  |  |  |
| Smoking dose^#^ |  |  |  |  |  |  |
| <5 cigarettes | 48.3% | (919) | 40.894 | 40.658 | 1.05 |  |
| 5-10 cigarettes | 27.7% | (528) | 36.695^b^ | 36.902 | 0.98 |  |
| >10 cigarettes | 24.0% | (457) | 34.106^c^ | 34.815^c^ | 0.92^c^ |  |
|  |  |  |  |  |  |  |
| Child Gender |  |  |  |  |  |  |
| Boy | 50.4% | (4062) | 38.326 | 38.363 | 1.05 |  |
| Girl | 49.6% | (4003) | 41.863^c^ | 41.825^c^ | 1.15^c^ |  |
|  |  |  |  |  |  |  |
| Education level |  |  |  |  |  |  |
| Low | 12.5% | (1013) | 38.622^a^ | 39.678^a^ | 1.11 |  |
| Middle (ref) | 47.2% | (3805) | 39.821 | 40.249 | 1.10 |  |
| High | 40.3% | (3247) | 40.841^c^ | 40.011 | 1.09 |  |
| ^a^ *P*<0.05 ; ^b^ *P*<0.01; ^c^ *P*<0.001 ; MoM = Multiple of gestational age specific median ; * multivariate analyses only. ^#^Smoking dosage was determined by the maximum self-reported number of cigarettes smoked during pregnancy and was not imputated due to poor imputation quality. Values shown as mean hCG in IU/L or MoM value in all women after exclusion of women with IVF treatment and twin or TOP pregnancies. Univariate analyses were adjusted for gestational age at blood sampling. Multivariate analyses were adjusted for gestational age at blood sampling, maternal age, parity, smoking, maternal education level, ethnicity, BMI and child gender. hCG/MoM values were log transformed for statistical analyses. | | | | | | |

| **Supplemental Table 5.** Mean hCG values according to characteristics of 8193 women from the Generation R study. | | | | | | | | |
| --- | --- | --- | --- | --- | --- | --- | --- | --- |
|  |  | | | **Mean hCG** (IU/L) | |  |  |  |
|  | **% or range (N)** | | | **Univariate** | **Multivariate** | **MoM hCG*** |  |  |
|  |  | | |  |  |  |  |  |
| **Fertility treatment** |  |  | |  |  |  |  |  |
| No | 98.7% | (7996) | | 39.287 | 40.083 | 1.10 |  |  |
| IVF | 0.5% | (38) | | 47.501^b^ | 47.740^a^ | 1.30^a^ |  |  |
| IUI | 0.1% | (11) | | 39.342 | 41.058 | 1.08 |  |  |
| Ovulation induction  ICSI or other | 0.7% | (58) | | 38.898 | 37.440 | 1.03 |  |  |
|  |  |  | |  |  |  |  |  |
| Twins |  | |  |  |  |  | |  |
| No | 99.0% | | (8065) | 40.099 | 40.096 | 1.10 | |  |
| Yes | 1.0% | | (82) | 67.488^c^ | 67.853^c^ | 1.83^c^ | |  |
| ^a^ *P*<0.05 ; ^b^ *P*<0.01; ^c^ *P*<0.001 ; MoM = Multiple of gestational age specific median ; * multivariate analyses only ;  Values shown as mean hCG in IU/L or MoM value in all women after exclusion of women with a TPO pregnancy and additionally IVF treatment or twin pregnancy, also, 8 women pregnant with twins after IVF treatment were excluded. Univariate analyses were adjusted for gestational age at blood sampling. Multivariate analyses were adjusted for gestational age at blood sampling, maternal age, parity, smoking, maternal education level, ethnicity, BMI and child gender. hCG/MoM values were log transformed for statistical analyses. | | | | | | | |  |

| **Supplemental Table 6.** Mean MoM hCG values according to self-reported episodes of hyperemesis gravidarum symptoms. | | | | | | |
| --- | --- | --- | --- | --- | --- | --- |
|  |  | | | **Mean MoM hCG** | |  |
|  | **% or range (N)** | | | **Univariate** | **Multivariate** |  |
|  |  | | |  |  |  |
| *Self-reported episodes of weekly* | | | |  |  |  |
| Reflux/belching |  | |  |  |  |  |
| Never (ref) | 44.6% | | (2998) | 1.08 | 1.08 |  |
| Less than once | 15.1% | | (1017) | 1.07 | 1.06 |  |
| Once | 9.2% | | (622) | 1.10 | 1.10 |  |
| Few times | 17.8% | | (1198) | 1.12^a^ | 1.12^b^ |  |
| Daily | 13.2% | | (890) | 1.16^c^ | 1.16^c^ |  |
| Linear β (SE) |  |  | | 0.017 (0.004); *P*<0.01 | 0.020 (0.004); *P*<0.01 |  |
|  |  | |  |  |  |  |
| Nausea |  | |  |  |  |  |
| Never (ref) | 18.5% | | (1263) | 1.03 | 1.05 |  |
| Less than once | 12.8% | | (871) | 1.09^b^ | 1.08 |  |
| Once | 8.2% | | (557) | 1.05 | 1.05 |  |
| Few times | 27.2% | | (1854) | 1.09^b^ | 1.09^b^ |  |
| Daily | 33.4% | | (2279) | 1.15^c^ | 1.14^c^ |  |
| Linear β (SE) |  | |  | 0.024 (0.004); *P*<0.01 | 0.021 (0.004); *P*=0.01 |  |
|  |  | |  |  |  |  |
| Vomiting |  | |  |  |  |  |
| Never (ref) | 55.2% | | (3696) | 1.08 | 1.08 |  |
| Less than once | 14.8% | | (991) | 1.09 | 1.08 |  |
| Once | 6.2% | | (418) | 1.10 | 1.10 |  |
| Few times | 13.2% | | (886) | 1.14^b^ | 1.14^b^ |  |
| Daily | 10.5% | | (706) | 1.13^a^ | 1.14^b^ |  |
| Linear β (SE) |  | |  | 0.015 (0.004); *P*<0.01 | 0.017 (0.004); *P*<0.01 |  |
| ^a^ *P*<0.05 ; ^b^ *P*<0.01; ^c^ *P*<0.001 ; MoM = Multiple of gestational age specific median. Values shown as mean MoM values in all women after exclusion of women with IVF treatment or twin pregnancy. Self-reported hyperemesis gravidarum symptoms were not imputated due to the large number of missing values. Univariate analyses were adjusted for gestational age at blood sampling. Multivariate analyses were adjusted for gestational age at blood sampling, maternal age, parity, smoking, maternal education level, ethnicity, BMI and child gender. MoM values were log transformed for statistical analyses. | | | | | | |
